# Relationships Between Self-Esteem and Personal Attributes, Income, Consumption, and Assets: Japanese Panel Study

**DOI:** 10.3390/ejihpe15050078

**Published:** 2025-05-12

**Authors:** Makoto Nakakita, Sakae Oya, Naoki Kubota, Tomoki Toyabe, Teruo Nakatsuma

**Affiliations:** 1 RIKEN Center for Advanced Intelligence Project, Tokyo 103-0027, Japan; 2 Centre for Finance, Technology and Economics at Keio, Keio University, Tokyo 108-8345, Japan; oya-econ@keio.jp; 3 Faculty of Economics, Keio University, Tokyo 108-8345, Japan; nk.kubota@keio.jp (N.K.); nakatuma@econ.keio.ac.jp (T.N.); 4 Kokushu Tech Innovation Platform, Tokyo 103-0013, Japan; 5 Faculty of Economics, Kanazawa Gakuin University, Ishikawa 920-1392, Japan; toyabe@kanazawa-gu.ac.jp

**Keywords:** self-esteem, socioeconomic factor, demographic factor, logit model, Bayesian estimation, Japan

## Abstract

Self-esteem is a key topic in psychology and health research. It influences well-being, happiness, and even medicine. However, existing studies on self-esteem have yielded conflicting results, suggesting that a global consensus remains elusive. This study examines how demographic and socioeconomic factors influence self-esteem in Japan. We analyzed panel data sampled from the entire Japanese population, with separate estimates performed for marital status and gender to account for potential differences in the impact of these factors. Using a Bayesian panel logit model with the Markov chain Monte Carlo method and the ancillarity–sufficiency interweaving strategy for efficient estimation, we found similarities and differences compared with studies from other countries. Furthermore, when comparing the overall data with data stratified by marital status and gender, we observed significant differences in how these factors influenced self-esteem, even among the same individuals. These findings underscore the importance of considering such variations when incorporating self-esteem into medical and healthcare contexts.

## 1. Introduction

Self-esteem is a key topic in psychology and healthcare research. It refers to how positively individuals perceive their own characteristics and qualities, as shaped by internal and external factors, including past successes and social evaluations. Closely linked to health and social well-being, high self-esteem supports positive mental health, reducing anxiety and stabilizing psychological function (Greenberg et al., 1992; Kernis, 2005; Liao et al., 2023; Liu et al., 2022; Pyszczynski et al., 2004; Yılmaz & Dundar, 2022) while lowering the risk of depression (Al Saigh et al., 2022; de la Torre-Cruz et al., 2021; Deng et al., 2020; Macinnes, 2006; Orth et al., 2009; Sowislo & Orth, 2013; Wang et al., 2018). Research has also linked self-esteem to illness acceptance and symptom relief (Colmsee et al., 2021; Fontaine & Jones, 1997; Gerymski & Szeląg, 2023), as well as stress reduction and tolerance (Gerymski et al., 2023; Wågan et al., 2021; Yıldırım et al., 2017). Additionally, self-esteem correlates positively with happiness and well-being (Baumeister et al., 2003; Lin, 2015; Pandey et al., 2019; Paradise & Kernis, 2002; Pignault et al., 2023; Trong Dam et al., 2023; Tu & Fu, 2024) along with life satisfaction (Gerymski et al., 2023; Kurnaz et al., 2020; Szcześniak et al., 2022; Trong Dam et al., 2023; Wodarz & Rogowska, 2024). It also benefits social relationships, academic success, and job performance (Auttama et al., 2021; Barragán Martín et al., 2021; Orth & Robins, 2022; Torres et al., 2022; Zapata-Lamana et al., 2021). Conversely, low self-esteem may contribute to criminal behavior, including violence and drug abuse (Albikawi, 2023; Boden et al., 2007; Donnellan et al., 2005; Hu et al., 2023; Trzesniewski et al., 2006), as well as self-harm and suicidality (Bhar et al., 2008; O’Connor et al., 2009; Soto-Sanz et al., 2019; Sunderland et al., 2020; Tatnell et al., 2014; Yoo et al., 2015). Moreover, a modern concern is the association between low self-esteem and internet, smartphone, and social media addiction, as observed in various countries, including Poland (Błachnio et al., 2016), Turkey (Aydm & San, 2011; Kircaburun, 2016), Iran (Bahrainian et al., 2014), Taiwan (H.-C. Chen et al., 2020), Greece (Gnardellis et al., 2023), and Norway (Steinsbekk et al., 2021). Casale et al. (2022) further confirmed this is as a worldwide trend.

Extensive research has examined the factors influencing self-esteem beyond its effects. von Soest et al. (2018) analyzed Norwegian adults and found that socioeconomic status, health, and social relationships significantly impact self-esteem, which peaks around age 50. McMullin and Cairney (2004) showed that gender, marital status, education, and income affect self-esteem among Canadians. Several studies have explored the role of financial wealth. For example, Rohe and Stegman (1994) found that homeownership does not influence self-esteem among low-income US adults, whereas housing conditions are an influencing factor. Truong and McColl (2011) reported that purchasing luxury goods boosts self-esteem among the French. Additionally, financial wealth benefits individuals and fosters their children’s self-esteem. Bannink et al. (2016) revealed that higher family income increased the likelihood of high self-esteem in 11-year-olds in the UK. Szkody et al. (2021) demonstrated that parenting styles received in childhood shape self-esteem in adulthood. Furthermore, gender differences are notable, with studies indicating that males generally have higher self-esteem compared with females (Bleidorn et al., 2016; Gentile et al., 2009; Josephs et al., 1992; McMullin & Cairney, 2004; Raymore et al., 1994; Rentzsch et al., 2016).

Research has also explored factors that diminish self-esteem. Although low self-esteem is linked to violence and crime, studies suggest that experiences such as bullying, violence, and sexual abuse from family members, partners, or friends can also reduce self-esteem (Bigizadeh et al., 2021; Cameranesi & Piotrowski, 2017; X. Chen et al., 2024; Jankowiak et al., 2021; Okunlola et al., 2020; Shen, 2008). Conversely, love and affection from family and friends can strengthen self-esteem (Luijten et al., 2023; Roberts & Bengtson, 1993; Shi et al., 2022). Other studies report that self-esteem reduces depression risk; however, depression itself can lower self-esteem (Gao et al., 2022; Shahar & Henrich, 2010). Beyond depression, physical and mental illnesses contribute to declining self-esteem (Auttama et al., 2021; Kalata et al., 2024).

Notably, self-esteem research often lacks consensus. Some studies report that high self-esteem negatively affects relationships and social adaptation (Baumeister et al., 2003; Valenti & Faraci, 2021). Opiri and Lang (2017) examined self-esteem’s relationship with consumption across various demographics, including race, age, and gender, concluding that it does not influence luxury brand purchases. Additionally, studies have shown that social media use does not impact self-esteem (Ma, 2022) and that gender differences in self-esteem do not appear among German elementary school students (Arens & Hasselhorn, 2014). Overall, the factors influencing self-esteem vary by region, race, and individual attributes, among other factors.

Previous studies revealed that the relationships of self-esteem with individual characteristics, such as age, sex, and socioeconomic status, can vary across cultural contexts. In particular, Japan differs from other countries in terms of social norms, values, and interpersonal expectations, which can influence the formation and expression of self-esteem. These cultural differences might affect the relationships of demographic and socioeconomic factors with self-esteem, suggesting that the mechanisms observed in other countries do not directly apply to the Japanese context. Therefore, it is important to analyze these associations within the cultural and societal framework of Japan. Examining self-esteem in this specific context enables a better understanding of its culturally embedded determinants, which might not be visible in studies based in other locations.

In this study, we investigate the relationship between self-esteem and multiple factors, including age, gender, marital status, region of residence, city size, personal and spousal income, consumption, and assets, focusing on the Japanese population. We also analyze the data separately by marital status and gender, given their impact on household finances and consumption. As self-esteem is shaped by individual characteristics that are not easily observed, we account for these differences by estimating individual effects in our model. Specifically, we use a Bayesian panel logit model to determine the presence of self-esteem, applying the Markov chain Monte Carlo (MCMC) method and the ancillarity–sufficiency interweaving strategy (ASIS) algorithm (Yu & Meng, 2011) for efficient parameter estimation. This study’s findings highlight the significant influence of geographical and cultural background on self-esteem, emphasizing the need to account for these differences when examining self-esteem in contexts such as medicine and healthcare.

The remainder of this paper is structured as follows. Section 2 details the materials and methods employed in the analysis, including the data source, variable construction, and modeling strategy. Section 3 presents the empirical results of the associations between self-esteem and a range of demographic and socioeconomic characteristics. Section 4 discusses the findings in relation to the existing literature, delineates the unique contributions of the present study, and critically examines its limitations. Section 5 summarizes the key insights of the study and suggests future research directions.

## 2. Materials and Methods

### 2.1. Data

This study used panel data from a 9-year questionnaire survey conducted in Japan from 2014 to 2022. The survey covered a broad range of topics, including family structure, personal attributes, education, employment, school attendance, activity time allocation, and cohabitation with parents. It was designed to reflect Japan’s overall population structure rather than a specific demographic, allowing for analysis of self-esteem across the general population. The survey data were provided by the Panel Data Research Center, Institute for Economic Studies, Keio University. Notably, each year’s survey was conducted in February, meaning that responses for a given year (e.g., 2020) reflect the previous year’s situation (e.g., 2019).

The data used in this study were obtained through a two-stage stratified random sampling process designed to ensure national representativeness. In the first stage, the country was stratified into 24 layers based on region and urban scales, and survey areas were allocated proportionally according to population size using the Basic Resident Register. Enumeration districts from the national census were randomly selected as survey units. In the second stage, approximately 10 individuals were randomly drawn from the resident registry of each selected area using systematic sampling. When originally selected participants were unavailable because of relocation, long-term absence, or nonresponse, pre-designated substitute individuals, who were matched by gender and age group within the same district, were used to maintain the planned sample size. This approach ensured that the sampling remained unbiased concerning gender and age.

The dataset included 39,250 responses from 5723 respondents over 9 years. However, not all respondents participated continuously. As this study estimated self-esteem as an unobservable individual effect, a stable number of responses per person was necessary for reliable estimation. Additionally, logit models incorporating individual effects require variation in responses, meaning that estimation is not possible if all responses from a respondent are identical (i.e., all ones or zeros). Therefore, we removed respondents meeting any of the following conditions:

- Condition 1: Fewer than five responses.
- Condition 2: All responses are ones or zeros.
- Condition 3: Residing outside Japan.

After applying these filters, the final dataset comprised 19,092 responses from 2280 respondents. As the data originated from a survey, missing values due to nonresponses or input errors were present. To address this, we applied the k-nearest neighbor algorithm, a machine learning method, to impute missing values.

Regarding variable selection, the survey’s broad scope allowed for a wide range of explanatory variables. Specifically, this study examined the effects of basic social attributes, residential areas, economic status, and life satisfaction on self-esteem. Table 1 and Table 2 detail the explanatory variables used. Self-esteem was defined as follows: if a respondent answered “Applicable” for the statement “I have something to live for”, they were classified as having self-esteem. Other response options included “Somewhat applicable”, “I cannot say either way”, “Not really applicable”, and “Inapplicable”. A binomial logit model was constructed, where 1 indicated self-esteem, and 0 indicated its absence.

**Figure 1:**
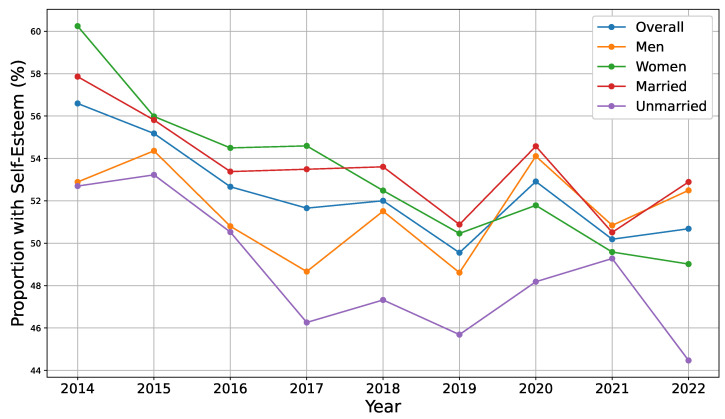
Proportion of individuals with self-esteem by group (2014–2022).

Figure 1 presents trends in self-esteem data from 2014 to 2022, including overall patterns and breakdowns by gender and marital status. The overall self-esteem level declined gradually from 2014 to 2019, rose slightly in 2020, and then fell sharply beginning in 2021. As the effects of the COVID-19 pandemic are reflected in the data from 2021 onward, the recent decline likely captures its psychological and social consequences. Married individuals consistently exhibited higher levels of self-esteem than unmarried individuals throughout the observation period, suggesting a stable association between marital status and psychological well-being. By contrast, a notable shift was observed in gender-based patterns. Namely, women reported higher self-esteem than men from 2014 to 2019, whereas this trend reversed in 2020, with men exhibiting higher levels for the remainder of the survey period. This reversal might reflect gendered differences in the effects of the pandemic and related social disruptions on individuals. Given the differences between men and women across various socioeconomic dimensions, such as employment patterns, caregiving responsibilities, and exposure to economic stressors, direct comparisons of self-esteem levels can be misleading without appropriate statistical controls. Our analysis highlighted the importance of adjusting for these covariates to better understand the independent relationships of gender and other factors with self-esteem in the Japanese context.

Although previous studies used various measures to assess self-esteem, standardized multiple-item scales–such as the Rosenberg Self-Esteem Scale (Rosenberg, 1965)–are among the most widely adopted. By contrast, this study defined self-esteem based on a single item: “I have something to live for”. Although this item reflects a related aspect of psychological well-being and does not capture the full scope of global self-esteem, it represents a meaningful indicator of perceived self-worth and life purpose. Moreover, previous research demonstrated that carefully selected single-item measures can offer valid insights in large-scale surveys, particularly when consistency across waves is required. Given the structure of our panel dataset, we adopted this item as a practical and interpretable proxy for self-esteem.

### 2.2. Model

We used Bayesian panel logit modeling with MCMC estimation to analyze the relationships between socioeconomic characteristics and psychological outcomes over time. This approach was particularly suitable for our panel data structure, as it allowed us to control for unobserved individual heterogeneity and derive probabilistic inferences through posterior distributions. Although recent advances in deep learning offer powerful tools for prediction, such methods often lack interpretability, and they are not designed for estimating the effects of specific covariates. Given our primary aim of drawing inference rather than maximizing prediction accuracy, the Bayesian framework offers both statistical rigor and substantive transparency that align with the goals of this study.

Below, we introduce the panel data logit model used in our analysis. Assuming that there are R=2280 respondents who completed questionnaires during the sample period, let r∈{1,…,R} be the index for each respondent, let $T_{r}$ be the number of times respondent *r* answered during this period, and let $t\in \{1,\ldots ,T_{r}\}$ denote the index for each annual questionnaire respondent *r* completed. The survey was conducted annually from 2014 to 2022; thus, $T_{r}$ ranges from 1 to 9. We define the following variables:

- $y_{rt}$: the binary response of respondent *r* in year *t*.
- $x_{rt}$: a K×1 vector of explanatory variables for respondent *r* in year *t*.

The response variable $y_{rt}$ is defined as follows:

$$y_{rt}=\left\{\begin{array}{c} 1,\;(\mathrm{Yes});\\ 0,\;(\mathrm{No}), \end{array}\right.\;r=1,\ldots ,R,\;t=1,\ldots ,T_{r}.$$

We also define

(1) $$\begin{array}{cc} y & ={\left[\begin{array}{c} y_{11}\ldots y_{1T_{1}}\ldots y_{R1}\ldots y_{RT_{R}} \end{array}\right]}^{\prime }, \end{array}$$

(2) $$\begin{array}{cc} X & ={\left[\begin{array}{c} x_{11}^{\prime }\ldots x_{1T_{1}}^{\prime }\ldots x_{R1}^{\prime }\ldots x_{RT_{R}}^{\prime } \end{array}\right]}^{\prime }. \end{array}$$

$y_{rt}$ is determined using the following equations:

(3) $$\begin{array}{cc} \; & y_{rt}∼\mathrm{Bernoulli}\left(\pi_{rt}\right), \end{array}$$

(4) $$\begin{array}{cc} \; & \pi_{rt}=P\left(y_{rt}=1|\psi_{rt}\right)=\frac{e^{\psi_{rt}}}{1+e^{\psi_{rt}}}, \end{array}$$

(5) $$\begin{array}{cc} \; & \psi_{rt}=\alpha_{r}+x_{rt}^{\prime }\beta . \end{array}$$

In these equations, $\alpha_{r}\;\left(r=1,\ldots ,R\right)$ is respondent *r*’s individual effect and β is a vector of regression coefficients for K×1. We then define

(6) $$\begin{array}{c} \alpha =\left[\begin{array}{c} \alpha_{1}\\ \vdots \\ \alpha_{R} \end{array}\right]. \end{array}$$

The likelihood function for the panel data logit model is expressed as follows:

(7) $$\begin{array}{cc} p(y|X,\alpha ,\beta ) & =\prod_{r=1}^{R}\prod_{t=1}^{T_{r}}\pi_{rt}^{y_{rt}}{\left(1-\pi_{rt}\right)}^{1-y_{rt}}\\ \; & =\prod_{r=1}^{R}\prod_{t=1}^{T_{r}}\frac{{\left(e^{\psi_{rt}}\right)}^{y_{rt}}}{1+e^{\psi_{rt}}}. \end{array}$$

We assume the following prior distribution for α and β:

(8) $$\alpha ∼N\left(\mu_{\alpha }\iota ,\sigma_{\alpha }^{2}I\right),\;\beta ∼N\left(\mu_{\beta },A_{\beta }^{-1}\right),$$

with the parameters in the hierarchical prior distribution of α defined as

(9) $$\begin{array}{c} \mu_{\alpha }∼N\left(\mu_{0},\sigma_{0}^{2}\right),\;\sigma_{\alpha }^{2}∼\mathrm{IG}\left(\frac{\nu_{0}}{2},\frac{\lambda_{0}}{2}\right), \end{array}$$

where IG(·) represents an inverse gamma distribution.

Applying Bayes’ theorem to the likelihood function in Equation (7) and prior distributions of the parameters in Equations (8) and (9), we obtain the posterior distribution:

(10) p(θ|D)∝p(y|X,α,β)p(θ),

where D=(y,X). Given that the posterior distribution in Equation (10) cannot be computed analytically, we employ the MCMC method. Specifically, we use Gibbs sampling (Geman & Geman, 1984) to estimate the parameters in Equation (10).

To facilitate efficient sampling in the panel data logit model, we use the Pólya–Gamma distribution:

(11) $$\begin{array}{cc} P & =\frac{1}{2\pi^{2}}\sum_{s=1}^{\infty }\frac{g_{s}}{{\left(s-\frac{1}{2}\right)}^{2}+\frac{c^{2}}{4\pi^{2}}}∼\mathrm{PG}\left(b,c\right), \end{array}$$

(12) $$\begin{array}{c} g_{s}∼Ga(b,1). \end{array}$$

According to Polson et al. (2013),

(13) $$\begin{array}{cc} \frac{{\left(e^{\psi }\right)}^{a}}{{\left(1+e^{\psi }\right)}^{b}} & =\int_{0}^{\infty }\frac{1}{2^{b}}e^{\kappa \psi -\frac{\omega \psi^{2}}{2}}p\left(\omega \right)d\omega , \end{array}$$

(14) $$\begin{array}{cc} \kappa =a-\frac{b}{2}, & \;\omega ∼\mathrm{PG}\left(b,0\right). \end{array}$$

Given that

(15) $$\frac{{\left(e^{\psi }\right)}^{a}}{{\left(1+e^{\psi }\right)}^{b}}=\left\{\begin{array}{c} \frac{e^{\psi }}{1+e^{\psi }}\;\left(a=1,b=1\right);\\ \frac{1}{1+e^{\psi }}\;\left(a=0,b=1\right), \end{array}\right.$$

and ω|ψ∼PG(b,ψ)(Polson et al., 2013), the full conditional of $\omega_{rt}$, considering $\psi_{rt}=\alpha_{r}+x_{rt}^{\prime }\beta$, is as follows:

(16) $$\omega_{rt}|y,X,\alpha ,\beta ∼\mathrm{PG}\left(1,\alpha_{r}+x_{rt}^{\prime }\beta \right).$$

Thus, the likelihood function in Equation (7) can be constructed as follows:

(17) $$\begin{array}{cc} p(y|X,\alpha ,\beta ) & =\prod_{r=1}^{R}\prod_{t=1}^{T_{r}}\int_{0}^{\infty }\frac{1}{2}\exp\left[\kappa_{rt}\psi_{rt}-\frac{\omega_{rt}\psi_{rt}^{2}}{2}\right]p\left(\omega_{rt}\right)d\omega_{rt}\\ & =\int_{0}^{\infty }\ldots \int_{0}^{\infty }p\left(y|X,\omega ,\alpha ,\beta \right)p\left(\omega_{11}\right)d\omega_{11}\ldots p\left(\omega_{RT_{R}}\right)d\omega_{RT_{R}}, \end{array}$$

(18) $$\begin{array}{cc} p(y|X,\omega ,\alpha ,\beta ) & =\prod_{r=1}^{R}\prod_{t=1}^{T_{r}}\exp\left[\kappa_{rt}\psi_{rt}-\frac{\omega_{rt}\psi_{rt}^{2}}{2}\right], \end{array}$$

where

(19) $$\begin{array}{cc} \omega & ={\left[\begin{array}{c} \omega_{11}\ldots \omega_{1T_{1}}\ldots \omega_{R1}\ldots \omega_{RT_{R}} \end{array}\right]}^{\prime }, \end{array}$$

(20) $$\begin{array}{cc} \kappa_{rt} & =y_{rt}-\frac{1}{2}. \end{array}$$

Consequently, we can write Equation (18) as follows:

(21) $$\begin{array}{cc} p(y|X,\omega ,\alpha ,\beta ) & =\prod_{r=1}^{R}\prod_{t=1}^{T_{r}}\frac{1}{2}\exp\left[\kappa_{rt}\left(\alpha_{r}+x_{rt}^{\prime }\beta \right)-\frac{\omega_{rt}}{2}{\left(\alpha_{r}+x_{rt}^{\prime }\beta \right)}^{2}\right]\\ \; & =2^{-RT}\exp\left[\sum_{r=1}^{R}\alpha_{r}\sum_{t=1}^{T_{r}}\kappa_{rt}+\sum_{r=1}^{R}\sum_{t=1}^{T_{r}}\kappa_{rt}x_{rt}^{\prime }\beta \right.-\frac{1}{2}\sum_{r=1}^{R}\alpha_{r}^{2}\sum_{t=1}^{T_{r}}\omega_{rt} \end{array}$$

(22) $$\begin{array}{cc} \; & \;-\sum_{r=1}^{R}\alpha_{r}\sum_{t=1}^{T_{r}}\omega_{rt}x_{rt}^{\prime }\beta \left.-\frac{1}{2}\sum_{r=1}^{R}\sum_{t=1}^{T_{r}}\omega_{rt}{\left(x_{rt}^{\prime }\beta \right)}^{2}\right], \end{array}$$

where $RT=\sum_{r=1}^{R}T_{r}$. Defining

(23) $$\begin{array}{cc} K & =\left[\begin{array}{c} \sum_{t=1}^{T_{1}}\kappa_{1t}\\ \vdots \\ \sum_{t=1}^{T_{R}}\kappa_{Rt} \end{array}\right],\;Z=\left[\begin{array}{c} \sum_{t=1}^{T_{1}}\omega_{1t}x_{1t}^{\prime }\\ \vdots \\ \sum_{t=1}^{T_{R}}\omega_{Rt}x_{Rt}^{\prime } \end{array}\right], \end{array}$$

(24) $$\begin{array}{cc} \bar{x} & =\sum_{r=1}^{R}\sum_{t=1}^{T_{r}}\kappa_{rt}x_{rt},\;\Omega =\mathrm{diag}\left(\omega \right),\;V=\mathrm{diag}\left\{\sum_{t=1}^{T_{1}}\omega_{1t},\ldots ,\sum_{t=1}^{T_{R}}\omega_{Rt}\right\}, \end{array}$$

we derive

(25) $$\begin{array}{c} p\left(y|X,\omega ,\alpha ,\beta \right)\propto \exp\left[K^{\prime }\alpha +{\bar{x}}^{\prime }\beta -\frac{1}{2}\alpha^{\prime }V\alpha -\alpha^{\prime }Z\beta -\frac{1}{2}\beta^{\prime }X^{\prime }\Omega X\beta \right]. \end{array}$$

Thus, the full conditionals of the parameters are as follows:

(26) $$\alpha |\theta_{-\alpha },D∼N\left({\left(V+\sigma_{\alpha }^{-2}I\right)}^{-1}(K-Z\beta +\mu_{\alpha }\sigma_{\alpha }^{-2}\iota ),{\left(V+\sigma_{\alpha }^{-2}I\right)}^{-1}\right),$$

(27) $$\beta |\theta_{-\beta },D∼N\left({\left(X^{\prime }\Omega X+A_{\beta }\right)}^{-1}\left(\bar{x}-Z^{\prime }\alpha +A_{\beta }\mu_{\beta }\right),{\left(X^{\prime }\Omega X+A_{\beta }\right)}^{-1}\right),$$

(28) $$\begin{array}{cc} \;\mu_{\alpha }|\theta_{-\mu_{\alpha }},D & ∼N\left(\frac{\sigma_{\alpha }^{2}\sum_{r=1}^{R}\alpha_{r}+\sigma_{0}^{-2}\mu_{0}}{\sigma_{\alpha }^{-2}R+\sigma_{0}^{-2}},\frac{1}{\sigma_{\alpha }^{-2}R+\sigma_{0}^{-2}}\right), \end{array}$$

(29) $$\begin{array}{cc} \;\sigma_{\alpha }^{2}|\theta_{-\sigma_{\alpha }^{2}},D & ∼\mathrm{IG}\left(\frac{R+\nu_{0}}{2},\frac{\sum_{r=1}^{R}{\left(\alpha_{r}-\mu_{\alpha }\right)}^{2}+\lambda_{0}}{2}\right). \end{array}$$

Using the full conditionals, we can estimate the posterior parameters numerically using the MCMC method. Although our proposed model is not highly complex, it involves estimating 2334 parameters based on a dataset of 19,092 observations, making it challenging to obtain stable estimates. To enhance computational efficiency, we employed the ASIS algorithm, a method that has been shown to improve parameter estimation in panel data models (e.g., Nakakita & Nakatsuma, 2024; Saito et al., 2024). For a detailed discussion of the derivation of full conditionals, the MCMC procedure, and the implementation of ASIS, refer to Appendix A.

To investigate the relationship between self-esteem and the explanatory variables, we first conducted a statistical analysis using the full dataset and all explanatory variables. Next, to assess whether the determinants of self-esteem vary by marital status and gender, we conducted two additional analyses: (1) stratifying the data by marital status and gender; (2) estimating a model with a reduced set of explanatory variables. The explanatory variables used in each model are shown in Table 3. Across all models, year dummies, age, and living region are included as control variables. Specifically, Model 1 incorporates all explanatory variables; Model 2 focuses on variables related to life satisfaction; and Model 3 emphasizes financial factors, such as savings, income, and expenditure. To determine the best-fitting model, we applied the widely applicable information criterion (WAIC) (Watanabe, 2010), a model selection metric suited for Bayesian estimation.

To perform Bayesian analysis, it is necessary to set the prior distribution. Thus, the prior distributions of $\left(\mu_{\beta },\Sigma_{\beta },\varphi_{\alpha },\tau_{\alpha }^{2},s_{\alpha },s_{\epsilon }\right)$ are given as follows:

(30) $$\begin{array}{cc} \mu_{\beta } & =0_{K},\;\Sigma_{\beta }=100I_{K},\\ \varphi_{\alpha } & =0,\;\tau_{\alpha }^{2}=100,\;s_{\alpha }=s_{\epsilon }=1. \end{array}$$

All models used the same prior distributions. Based on the settings above, all statistical analyses were conducted using Python 3.13.1. The computations relied on the following libraries: NumPy (v2.2.2), SciPy (v1.15.1), and Polyagamma (v2.0.1).

## 3. Results

**Figure 2:**
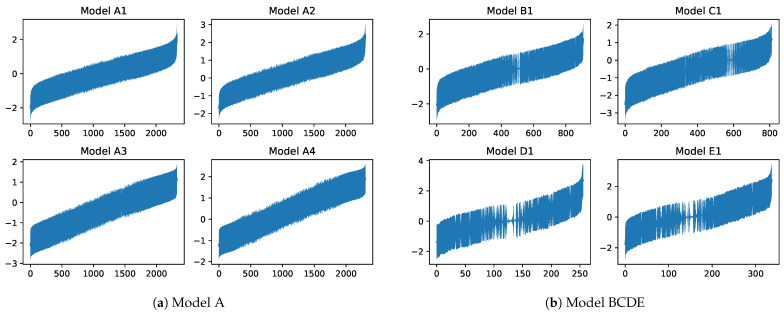
Plots of individual effects on respondents’ self-esteem, ordered from lowest to highest. Bold lines represent the posterior means, and their widths indicate ±1 posterior standard deviations.

Below, we present the estimation results using different datasets and models, along with interpretations and comparisons. The results are summarized in Figure 2 as well as Table 4 and Table 5a–d.

### 3.1. Estimation Results Using Data from All Respondents

We initially examined the estimation results based on data from all respondents, as shown in Table 4. According to the WAIC, Model A1, which included all explanatory variables, was the best-fitting model. Therefore, we focused on analyzing its regression coefficients in detail.

The coefficient for having a spouse, $\beta_{a}$, was positive, with a posterior mean of 0.207, indicating a positive association with self-esteem. In contrast, the male dummy coefficient, $\beta_{b}$, was −0.146, suggesting that men tend to have lower self-esteem compared with women. Notably, the effect of having a spouse was relatively larger in magnitude than the effect of gender. Regarding year dummy coefficients, all posterior means were negative, and except for $\beta_{1,1}$, their 95% confidence intervals did not include zero. Additionally, the absolute values of these coefficients tended to increase over time, indicating a steady decline in self-esteem among Japanese people. Considering the large negative values of $\beta_{1,5},x_{1,7},\;\mathrm{and}\;\beta_{1,8}$, the $\beta_{1,7}$ and $\beta_{1,8}$ values may have reflected psychological effects of the COVID-19 pandemic. Although $\beta_{1,8}$ corresponded to 2019, the survey conducted in February captured conditions from 2018. The large negative coefficient for $\beta_{1,5}$ may have been linked to a series of major natural disasters in 2018, including a magnitude 6.1 earthquake in Osaka (the second largest city in Japan) in June, the West Japan floods (resulting in over 220 deaths) in July, and the magnitude 7 Hokkaido earthquake (causing a blackout across the area) in September. These events likely contributed to economic damage as well as a sense of helplessness, which may have negatively affected self-esteem. Examining the age dummy coefficients, $\left(\beta_{2,1},\ldots ,\beta_{2,4}\right)$, all posterior means were negative, and all except for $\beta_{2,1}$ were significant at the 5% level. Furthermore, self-esteem tended to decline as age increased from $\beta_{2,1}$ to $\beta_{2,4}$. In terms of regional ($\beta_{3,1},\ldots ,\beta_{3,7}$) and city size ($\beta_{4,1},\beta_{4,2}$) dummies, the posterior means varied between positive and negative, but their standard deviations were relatively large, suggesting that neither region nor city size had a strong effect on self-esteem.

Next, we analyzed the effects of life satisfaction variables ($\beta_{5,1},\ldots ,\beta_{5,7}$) on self-esteem. Household income satisfaction, $\beta_{5,1}$, had a significantly negative coefficient, which seemed counterintuitive. However, it was possible that individuals with high self-esteem set higher personal standards, leading to lower satisfaction with their income despite earning more. The coefficient for employment satisfaction, $\beta_{5,2}$, was positive, indicating that job satisfaction was associated with higher self-esteem. Housing satisfaction, $\beta_{5,3}$, appeared to have no relationship with self-esteem. For leisure-related variables, the amount and quality of leisure time, $\beta_{5,4}\;\mathrm{and}\;\beta_{5,5}$, showed negative and positive regression coefficients, respectively. The former suggests that excessive leisure time may have reduced self-esteem, possibly due to a lack of meaningful engagement. The latter implies that taking part in fulfilling leisure experiences, in terms of time and relationships, boosts self-esteem. Health satisfaction, $\beta_{5,6}$, also had a positive coefficient, indicating a positive effect on self-esteem, consistent with prior studies (as mentioned in Section 1). Finally, overall life satisfaction, $\beta_{5,7}$, had the most substantial positive coefficient among the satisfaction variables, suggesting that general life contentment played the most significant role in shaping self-esteem.

We then considered the regression coefficients of the quantitative explanatory variables related to economic factors, such as assets, income, and consumption. The coefficients $\beta_{6,1},\ldots ,\beta_{6,5}$ represent the effects of household assets, income, and debt on self-esteem. Only the regression coefficient for debt ($\beta_{6,3}$), which was positive, was significant. This may indicate that individuals who borrowed more were more active in their economic activities, or that those with high self-esteem tended to borrow more, feeling confident about their ability to repay. The effects of income from the respondent, their spouse, and other family members ($\beta_{7,1},\ldots ,\beta_{7,7}$) were all significant, with the signs varying from positive to negative. These results suggest that the amount of income itself did not have a direct effect on self-esteem. Next, we examined the effects of expenditures ($\beta_{8,1},\ldots ,\beta_{8,6}$), all of which were positive, with $\beta_{8,2}$ (covering rent, land rent, and home repairs) showing a significant result. This suggests that individuals with higher rent costs, who are, as such, more invested in their homes, tend to have higher self-esteem. Notably, expenditures appeared more closely related to self-esteem than to income among Japanese people. Regarding loan expenditure ($\beta_{9,1}$), the coefficient was significantly negative. This contrasts with the positive coefficient for debt, $\beta_{6,3}$, as $\beta_{6,3}$ reflects total debt, whereas $\beta_{9,1}$ reflects annual loan repayments. Loan repayment increases awareness of debt, which may lead to lower self-esteem.

The coefficient for donations ($\beta_{9,2}$) was positive and significant, indicating that larger donations, as a charitable activity and a form of societal contribution, were associated with higher self-esteem. Finally, the effects of housing and land asset values ($\beta_{10,1}$ and $\beta_{10,2}$) were not significant, similar to the lack of correlation between savings/securities and self-esteem. These results suggest that Japanese people’s self-esteem is not influenced by the assets they own.

The above findings were from Model A1. Notably, Table 4 shows no clear differences among the results of all models. Therefore, despite Model A1 being the best model, the effects of explanatory variables on self-esteem were similar across other models, indicating robust results. Considering Figure 2a, which shows individual effects in ascending order, the central line and bands represent the posterior mean and posterior standard deviation (±1 standard deviation), respectively. The results were consistent across all models, with the difference between the largest and smallest effects being approximately 4. The effect of having a spouse (0.207) and the effect of gender (−0.146) showed that individual characteristics had a substantial impact on self-esteem. This reinforces the importance of controlling for individual effects in social and economic analyses.

Given the large dataset, including 19,092 responses with 2334 parameters, stable estimation was challenging. However, by applying ASIS and other techniques, the Gelman–Rubin statistics for all parameters were below 1.01, indicating successful sampling convergence. Such efficient estimation is a key aspect of this study.

### 3.2. Estimation Results with Data Stratified by Marital Status and Gender

We also examined results for data separated by marital status and gender, as shown in Table 4 and Table 5a–d. According to the WAIC, the models with “1” in all cases (B, C, D, and E) were the best (Table 4); therefore, we focused on these results.

A key difference was observed in Model A1 for the year dummy ($\beta_{1,1},\ldots ,\beta_{1,8}$). In this model, all year dummies were negative and mostly significant, whereas in Model B1, the coefficients had mixed signs and were less significant. Model D1, which was identical to Model B1 except that it only included males, showed similar mixed signs. In contrast, Model C1, akin to Model A1, had all negative coefficients, most of which were significant. In Model E1, which was focused only on females, the significance of the coefficients decreased, but all signs remained negative. Notably, the effects of years involving the COVID-19 pandemic ($\beta_{1,7}$ and $\beta_{1,8}$) were negative for women but either nonsignificant or positive for men. This suggests that women’s self-esteem changed year by year, whereas men’s self-esteem remained unaffected by specific events, such as the pandemic.

The age dummies ($\beta_{2,1},\ldots ,\beta_{2,4}$) varied by marital status. In models for married individuals (Models B1 and C1), the coefficients were generally significantly negative, with posterior means decreasing over time. In contrast, for unmarried individuals (Models D1 and E1), none of the age dummies were significant, and for unmarried men, all posterior means were positive except for one. This indicates that married individuals experienced a decline in self-esteem with age, whereas unmarried individuals did not. These results highlight the differences in how marital status or gender influence the factors affecting self-esteem. Specifically, married individuals’ self-esteem decreased with age, whereas unmarried individuals, especially men, did not experience the same trend. This finding is a key insight of our research.

Regarding region dummies ($\beta_{3,1},\ldots ,\beta_{3,7}$), a notable change from Model A1 was observed in $\beta_{3,4}$ for Models B1 and D1 (men only), where the values shifted significantly to positive. The Kinki region ($\beta_{3,4}$), which includes Osaka and Kyoto (a city with a rich history and tradition), is economically and culturally prosperous, suggesting that living in such areas may positively affect men’s self-esteem. Finally, city size dummies ($\beta_{4,1}$ and $\beta_{4,2}$) were not significant in any model, indicating that city size did not influence self-esteem in Japanese people.

The results for satisfaction levels ($\beta_{5,1},\ldots ,\beta_{5,7}$) were generally similar for Model A1 and the other models. The coefficients for satisfaction with employment ($\beta_{5,2}$) and life overall ($\beta_{5,7}$) were positive in all models, showing a consistent positive relationship with self-esteem regardless of marital status or gender. Satisfaction with leisure time ($\beta_{5,4}$) had a negative effect on self-esteem for those with a spouse but no effect for those without a spouse. Satisfaction with the quality of leisure time ($\beta_{5,5}$) was positive for married men and women, as well as women without a spouse, whereas men without a spouse exhibited no relationship between leisure time and self-esteem. These results suggest that attitudes toward leisure may vary by marital status and gender.

For assets, borrowing, income, and expenditure, most effects were not significant, as in Model A1. However, notable results included a significantly positive effect of household savings ($\beta_{6,1}$) in Model B1. In Japan, where men typically work and women usually manage the home, this result may reflect the role of men as primary earners and savers, particularly for family expenses, such as family support and children’s education.

In Model C1, the effects of borrowing ($\beta_{6,3}$) and donations ($\beta_{9,2}$) were significantly positive. These results, consistent with Model A1, along with Model E1 for $\beta_{6,3}$, suggest that married women’s willingness to borrow and donate is positively related to their self-esteem. In Model D1, the coefficient for other income ($\beta_{7,3}$) was significantly negative. Other income, such as money obtained from relatives, public pensions, and unemployment benefits, among other sources, had a negative effect on unmarried men’s self-esteem. Although the coefficient of $\beta_{7,3}$ in Model E1 was nonsignificantly negative, in Models B1 and C1 it was nonsignificantly positive. This highlights the differing values and characteristics of other income based on marital status. An interesting finding in Model E1 was the positive effect of securities ($\beta_{6,2}$) alongside borrowing ($\beta_{6,3}$), suggesting that financial risk taking and financial assets are positively associated with self-esteem.

Next, we examined individual effects. As shown in Figure 2b, the overall distributions in Models B1–E1 were similar to that in Model A1. However, Models D1 and E1 had more individuals with small posterior standard deviations, indicating less variance in individual effects from year to year when analyzed by marital status and gender. This approach allowed for more precise estimation of individual effects compared with the analysis of all data together.

## 4. Discussion

Self-esteem is a crucial topic in psychology and healthcare. It influences well-being, happiness, illness recovery, and even the reduction of criminal impulses. However, research on self-esteem has yielded varying results across regions and individual characteristics, preventing a global consensus. This study addressed this gap using household panel survey data representative of the Japanese society to identify and quantify factors influencing self-esteem in Japan. Our findings provide a quantitative understanding of how demographic and socioeconomic attributes relate to self-esteem, revealing some inconsistencies with prior literature and highlighting the contextual nature of self-esteem.

Considering the differences between our findings and those of previous research, we first address the effect of gender on self-esteem. Most studies have shown that men have higher self-esteem compared with women (e.g., Bleidorn et al., 2016; Gentile et al., 2009; Josephs et al., 1992; McMullin & Cairney, 2004; Raymore et al., 1994; Rentzsch et al., 2016). However, we found that the opposite was true in Japan. This may stem from cultural differences or the inclusion of individual effects and various socioeconomic variables in our analysis, which may have provided a more accurate assessment of the gender–self-esteem relationship. Regardless, given that our data reflect Japan’s population structure, it is reasonable to conclude that self-esteem is generally higher in Japanese women than in Japanese men. Age-related trends also differed in our study relative to previous research. von Soest et al. (2018) found that self-esteem among Norwegians peaks at age 50, whereas our results show a peak in the 30 s, followed by a decline. Housing satisfaction also produced differing results. Although Rohe and Stegman (1994) found that housing conditions positively affect self-esteem in the USA, we observed no relationship in Japan, except for in married men. These findings suggest fundamental differences in attitudes toward housing between Japan and the USA. However, our results align with prior studies regarding the relationship between health and self-esteem, indicating that although some aspects of self-esteem are culturally related, others are more universal.

Cross-cultural research further supports the notion that self-esteem functions differently in Japan. Colwell and Kato (2003), comparing Japan and the UK, found no link between gaming and self-esteem among Japanese adolescents. Radford et al. (1993) reported that Japanese students had lower decisional self-esteem compared with their Australian counterparts. Hofer et al. (2016) found that patients with schizophrenia in Japan had lower resilience and self-esteem but higher hopelessness relative to Austrian patients. Szeto et al. (2009) reported significantly lower implicit self-esteem in Japanese people compared with Canadians. These findings suggest that cultural and environmental factors shape self-esteem differently in Japan relative to other regions. Our study reinforces this by showing that even within Japan, self-esteem determinants vary significantly by marital status and gender. Given this variability, it is unsurprising that global self-esteem research yields inconsistent results. Nonetheless, as highlighted in Section 1, self-esteem is generally considered beneficial, and our findings provide valuable insights into the factors influencing self-esteem in Japan.

Another potential reason for the discrepancies with previous studies is measurement differences. Many studies have used Rosenberg’s self-esteem scale as a measure of self-esteem, whereas we used responses to the survey item “I have something to live for”. As our dataset was not specifically designed to measure self-esteem, our definition may not fully align with that employed in previous research. However, our dataset’s size and breadth, which covered demographic information, employment behavior, poverty dynamics, and real asset transfers, allowed us to measure factor effects while controlling for a higher number of variables relative to prior studies. Furthermore, by leveraging panel data, we accounted for unobservable individual characteristics and effects, strengthening our analysis. Despite these advantages, a key limitation is our study’s inability to establish causal relationships between self-esteem and explanatory variables. Future research should explore causal links using time-series analysis with panel data.

Despite the strengths of this study, it has several limitations, which should be acknowledged. First, our analysis relied on self-reported measures of self-esteem and socioeconomic factors, which can be subject to social desirability bias or measurement error. Second, although we used panel data to capture changes over time, the observational nature of the study limited our ability to draw causal conclusions. Third, the possibility of unobserved confounding variables that influenced self-esteem and the explanatory factors, for which we could not fully control, must be acknowledged. Finally, as the study focused solely on the Japanese population, the findings might not be directly generalizable to other cultural contexts. Nevertheless, the cultural specificity of our analysis is also a strength, offering valuable insight into the contextual determinants of self-esteem in Japan.

Despite these limitations, our findings offer valuable insights for policy development in Japan. The observed higher self-esteem among women and married individuals suggests that psychological counseling and health interventions can benefit from targeted approaches that consider gender and marital status. Furthermore, our analysis of additional factors such as age, education level, and employment status indicates that a multifaceted strategy addressing various demographic variables could enhance the effectiveness of such interventions. By tailoring programs to the specific needs of different demographic groups, policymakers can design more effective mental health support systems that resonate with the diverse experiences of the Japanese population.

## 5. Conclusions

We analyzed the relationships between demographic and socioeconomic factors, such as income, consumption, and assets, and self-esteem in Japanese people. Recognizing that these relationships may vary by marital status and gender, we conducted an overall analysis and separate estimations for different subgroups stratified accordingly. Using a Bayesian panel logit model estimated via MCMC with ASIS for efficiency, we identified similarities and differences between our findings and those of related studies in other countries. Notably, even within the Japanese population, the impact of various factors on self-esteem differed markedly based on individual attributes. These findings highlight the influence of geographical and cultural context on self-esteem, emphasizing the need to account for such differences when applying self-esteem research in fields such as medicine and healthcare. Future research should explore causal relationships between self-esteem and demographic or socioeconomic factors using longitudinal methods and advanced statistical techniques. Additionally, multinational studies could help clarify the influence of cultural norms and societal structures on the formation and expression of self-esteem. Further investigation into the psychological mechanisms underlying observed associations would also contribute to a more comprehensive understanding of self-esteem across diverse contexts.

## Figures and Tables

**Table 1:** Descriptive statistics for explained and dummy variables.

		Frequency		Mean	
Variable	Description	1	0	1	0
Self-esteem	Having self-esteem (default: No)				
*y*	Yes	10,021	9071	1	0
Spouse	Having a spouse (default: No)				
$x_{a}$	Yes	14,240	4852	0.538	0.487
Sex	(default: Female)				
$x_{b}$	Male	9373	9719	0.516	0.534
Year	(default: 2014)				
$x_{1,1}$	2015	2269	16,823	0.552	0.521
$x_{1,2}$	2016	2271	16,821	0.527	0.525
$x_{1,3}$	2017	2265	16,827	0.517	0.526
$x_{1,4}$	2018	2271	16,821	0.520	0.526
$x_{1,5}$	2019	2129	16,963	0.496	0.529
$x_{1,6}$	2020	2009	17,083	0.529	0.524
$x_{1,7}$	2021	1853	16,239	0.502	0.527
$x_{1,8}$	2022	1758	17,334	0.507	0.527
Age	(default: –39)				
$x_{2,1}$	40–49	4121	14,971	0.568	0.513
$x_{2,2}$	50–59	4324	14,768	0.516	0.527
$x_{2,3}$	60–69	4240	14,852	0.492	0.534
$x_{2,4}$	70–	4322	14,770	0.500	0.532
Region	Region in which respondent resides (default: Kanto)				
$x_{3,1}$	Hokkaido	912	18,180	0.508	0.528
$x_{3,2}$	Tohoku	1226	17,866	0.502	0.526
$x_{3,3}$	Chubu	3358	15,734	0.509	0.528
$x_{3,4}$	Kinki	3521	15,571	0.538	0.522
$x_{3,5}$	Chugoku	1195	17,897	0.519	0.525
$x_{3,6}$	Shikoku	518	18,574	0.475	0.526
$x_{3,7}$	Kyushu	2052	17,040	0.545	0.522
Size of city	Size of city in which respondent resides (default: Major [government-designated]				
$x_{4,1}$	Other cities	11,578	7514	0.520	0.533
$x_{4,2}$	Towns and villages	1791	17,301	0.523	0.525

**Table 2:** Descriptive statistics for quantitative variables.

Variable	Description	Mean	SD	Median	Max	Min
Satisfaction level						
$x_{5,1}$	Household income	−0.386	2.458	0	5	−5
$x_{5,2}$	Employment status	0.157	2.336	0	5	−5
$x_{5,3}$	Housing	0.939	2.328	0	5	−5
$x_{5,4}$	Amount of leisure time	0.582	2.349	0	5	−5
$x_{5,5}$	Leisure time activities	0.547	2.208	0	5	−5
$x_{5,6}$	Health	0.478	2.198	0	5	−5
$x_{5,7}$	Life overall	0.798	1.931	0	5	−5
Household finances						
$x_{6,1}$	Amount of savings and deposits	4.789	2.989	5.920	10.309	0
$x_{6,2}$	Amount of securities	1.304	2.522	0	10.021	0
$x_{6,3}$	Borrowings	2.525	3.264	0	10.820	0
$x_{6,4}$	Household’s annual income	6.250	0.737	6.347	9.210	0
$x_{6,5}$	Household’s asset income	0.552	1.518	0	9.306	0
Individual income						
$x_{7,1}$	Respondent’s business and salary income	4.847	2.381	5.861	8.987	0
$x_{7,2}$	Respondent’s investment income	0.592	1.483	0	9.210	0
$x_{7,3}$	Respondent’s other income	2.452	2.507	2.398	7.645	0
$x_{7,4}$	Spouse’s business and salary income	2.340	2.484	0	7.824	0
$x_{7,5}$	Spouse’s investment income	0.176	0.799	0	7.456	0
$x_{7,6}$	Spouse’s other income	1.277	1.950	0	7.700	0
$x_{7,7}$	Other family members’ total income	2.635	2.810	0	8.638	0
Household consumption						
$x_{8,1}$	Food, eating out, and school lunches	4.244	0.523	4.262	6.553	0
$x_{8,2}$	Rent, land rent, home repairs	1.449	1.904	0	8.700	0
$x_{8,3}$	Furniture and digital consumer electronics purchases	1.192	1.506	0	8.189	0
$x_{8,4}$	Clothing and shoes	1.995	1.278	2.398	6.547	0
$x_{8,5}$	Education, culture, and amusement	2.035	1.656	2.398	8.061	0
$x_{8,6}$	Other expenditures	4.580	0.884	4.700	8.320	0
Other expenses						
$x_{9,1}$	Loan repayments	0.964	1.177	0	6.804	0
$x_{9,2}$	Donations to charity and religious organizations	2.121	3.841	0	14.988	0
Market value of the house and the plot						
$x_{32,1}$	House’s market value	7.930	5.438	6.909	16.118	0
$x_{32,2}$	Plot’s market value	7.979	4.593	7.314	13.816	0

Responses regarding satisfaction are centered, with the value corresponding to “neither satisfied nor dissatisfied” being zero. Satisfaction is measured on a scale from dissatisfied (−5) to satisfied (5). All monetary responses other than $x_{9,2}$ are in units of 10 thousand yen. Variables were generated by applying the logarithm to the original response after adding one.

**Table 3:** Models and explanatory variables used in the analyses.

Variable	Model 1	Model 2	Model 3	Model 4
Year				
$x_{1,1}$	✓	✓	✓	✓
$x_{1,2}$	✓	✓	✓	✓
$x_{1,3}$	✓	✓	✓	✓
$x_{1,4}$	✓	✓	✓	✓
$x_{1,5}$	✓	✓	✓	✓
$x_{1,6}$	✓	✓	✓	✓
$x_{1,7}$	✓	✓	✓	✓
$x_{1,8}$	✓	✓	✓	✓
Age				
$x_{2,1}$	✓	✓	✓	✓
$x_{2,2}$	✓	✓	✓	✓
$x_{2,3}$	✓	✓	✓	✓
$x_{2,4}$	✓	✓	✓	✓
Region				
$x_{3,1}$	✓	✓	✓	✓
$x_{3,2}$	✓	✓	✓	✓
$x_{3,3}$	✓	✓	✓	✓
$x_{3,4}$	✓	✓	✓	✓
$x_{3,5}$	✓	✓	✓	✓
$x_{3,6}$	✓	✓	✓	✓
$x_{3,7}$	✓	✓	✓	✓
City size				
$x_{4,1}$	✓	✓	✓	✓
$x_{4,2}$	✓	✓	✓	✓
Satisfaction level				
$x_{5,1}$	✓	✓		
$x_{5,2}$	✓	✓		
$x_{5,3}$	✓	✓		
$x_{5,4}$	✓	✓		
$x_{5,5}$	✓	✓		
$x_{5,6}$	✓	✓		
$x_{5,7}$	✓	✓		
Household finances				
$x_{6,1}$	✓		✓	
$x_{6,2}$	✓		✓	
$x_{6,3}$	✓		✓	
$x_{6,4}$	✓		✓	
$x_{6,5}$	✓		✓	
Individual income				
$x_{7,1}$	✓		✓	
$x_{7,2}$	✓		✓	
$x_{7,3}$	✓		✓	
$x_{7,4}$	✓		✓	
$x_{7,5}$	✓		✓	
$x_{7,6}$	✓		✓	
$x_{7,7}$	✓		✓	
Household consumption				
$x_{8,1}$	✓		✓	
$x_{8,2}$	✓		✓	
$x_{8,3}$	✓		✓	
$x_{8,4}$	✓		✓	
$x_{8,5}$	✓		✓	
$x_{8,6}$	✓		✓	
Other expenses				
$x_{9,1}$	✓		✓	
$x_{9,2}$	✓		✓	
House and plot market value				
$x_{32,1}$	✓		✓	
$x_{32,2}$	✓		✓	

**Table 4:** Posterior means and posterior standard deviations estimated using all respondents’ data.

β	Model A1	Model A2	Model A3	Model A4
$\beta_{a}$	0.207(0.080)	0.274(0.061)	0.146(0.079)	0.297(0.061)
$\beta_{b}$	−0.146(0.054)	−0.141(0.055)	−0.132(0.055)	−0.122(0.056)
$\beta_{1,1}$	−0.020(0.070)	−0.023(0.068)	−0.061(0.067)	−0.063(0.067)
$\beta_{1,2}$	−0.141(0.069)	−0.153(0.069)	−0.172(0.067)	−0.180(0.067)
$\beta_{1,3}$	−0.179(0.069)	−0.192(0.069)	−0.213(0.067)	−0.223(0.067)
$\beta_{1,4}$	−0.180(0.073)	−0.192(0.069)	−0.187(0.071)	−0.198(0.067)
$\beta_{1,5}$	−0.322(0.075)	−0.330(0.070)	−0.311(0.073)	−0.315(0.069)
$\beta_{1,6}$	−0.191(0.075)	−0.201(0.073)	−0.133(0.073)	−0.138(0.070)
$\beta_{1,7}$	−0.254(0.078)	−0.290(0.075)	−0.235(0.076)	−0.263(0.073)
$\beta_{1,8}$	−0.252(0.080)	−0.270(0.076)	−0.227(0.077)	−0.242(0.073)
$\beta_{2,1}$	−0.088(0.082)	−0.054(0.083)	−0.117(0.083)	−0.068(0.082)
$\beta_{2,2}$	−0.309(0.089)	−0.291(0.090)	−0.310(0.089)	−0.266(0.087)
$\beta_{2,3}$	−0.428(0.096)	−0.469(0.092)	−0.334(0.097)	−0.355(0.090)
$\beta_{2,4}$	−0.432(0.108)	−0.517(0.094)	−0.257(0.107)	−0.348(0.092)
$\beta_{3,1}$	−0.073(0.134)	−0.098(0.133)	−0.014(0.136)	−0.070(0.138)
$\beta_{3,2}$	0.114(0.119)	0.064(0.119)	0.019(0.122)	−0.091(0.122)
$\beta_{3,3}$	−0.046(0.080)	−0.064(0.079)	−0.055(0.082)	−0.074(0.081)
$\beta_{3,4}$	0.096(0.077)	0.095(0.078)	0.082(0.080)	0.071(0.080)
$\beta_{3,5}$	−0.011(0.115)	−0.022(0.117)	−0.029(0.118)	−0.039(0.120)
$\beta_{3,6}$	0.016(0.169)	−0.040(0.169)	−0.132(0.175)	−0.209(0.167)
$\beta_{3,7}$	0.088(0.094)	0.052(0.097)	0.174(0.098)	0.104(0.098)
$\beta_{4,1}$	−0.075(0.061)	−0.080(0.061)	−0.059(0.061)	−0.062(0.062)
$\beta_{4,2}$	0.004(0.105)	0.016(0.103)	−0.012(0.107)	0.015(0.106)
$\beta_{5,1}$	−0.031(0.011)	−0.028(0.011)		
$\beta_{5,2}$	0.115(0.010)	0.115(0.010)		
$\beta_{5,3}$	0.002(0.011)	0.005(0.011)		
$\beta_{5,4}$	−0.075(0.013)	−0.076(0.013)		
$\beta_{5,5}$	0.110(0.014)	0.107(0.014)		
$\beta_{5,6}$	0.026(0.012)	0.025(0.012)		
$\beta_{5,7}$	0.252(0.018)	0.256(0.012)		
$\beta_{6,1}$	0.014(0.009)		0.033(0.008)	
$\beta_{6,2}$	0.008(0.010)		0.012(0.010)	
$\beta_{6,3}$	0.026(0.008)		0.024(0.008)	
$\beta_{6,4}$	−0.019(0.038)		0.065(0.037)	
$\beta_{6,5}$	0.025(0.013)		0.026(0.013)	
$\beta_{7,1}$	0.010(0.012)		0.021(0.012)	
$\beta_{7,2}$	−0.011(0.016)		−0.001(0.016)	
$\beta_{7,3}$	0.006(0.012)		0.014(0.012)	
$\beta_{7,4}$	0.007(0.011)		0.012(0.011)	
$\beta_{7,5}$	0.032(0.028)		0.044(0.028)	
$\beta_{7,6}$	−0.024(0.015)		−0.027(0.015)	
$\beta_{7,7}$	−0.009(0.008)		−0.013(0.008)	
$\beta_{8,1}$	0.002(0.045)		−0.018(0.045)	
$\beta_{8,2}$	0.025(0.012)		0.015(0.011)	
$\beta_{8,3}$	0.001(0.013)		−0.004(0.012)	
$\beta_{8,4}$	0.003(0.017)		0.010(0.016)	
$\beta_{8,5}$	0.023(0.013)		0.034(0.013)	
$\beta_{8,6}$	0.038(0.023)		0.038(0.023)	
$\beta_{9,1}$	−0.005(0.019)		−0.017(0.019)	
$\beta_{9,2}$	0.021(0.006)		0.018(0.005)	
$\beta_{10,1}$	−0.003(0.004)		−0.002(0.004)	
$\beta_{10,2}$	−0.006(0.005)		−0.007(0.004)	
$\mu_{\alpha }$	−0.045(0.287)	0.244(0.109)	−0.419(0.279)	0.411(0.109)
$\sigma_{\alpha }$	0.982(0.054)	1.007(0.056)	1.087(0.057)	1.132(0.059)
WAIC	**11,370.0**	11,488.4	14,773.1	14,944.7

**Table 5:** Posterior means and posterior standard deviations by models and data types. (**a**) Model B (men, married). (**b**) Model C (women, married). (**c**) Model D (men, unmarried). (**d**) Model E (women, unmarried).

(a)				
β	**Model B1**	**Model B2**	**Model B3**	**Model B4**
$\beta_{1,1}$	0.127(0.113)	0.118(0.113)	0.074(0.109)	0.072(0.109)
$\beta_{1,2}$	0.015(0.113)	−0.003(0.113)	−0.051(0.110)	−0.062(0.109)
$\beta_{1,3}$	−0.062(0.113)	−0.077(0.114)	−0.097(0.109)	−0.109(0.108)
$\beta_{1,4}$	0.090(0.120)	0.039(0.113)	0.068(0.115)	0.023(0.110)
$\beta_{1,5}$	−0.015(0.121)	−0.056(0.116)	−0.060(0.115)	−0.092(0.113)
$\beta_{1,6}$	0.240(0.125)	0.196(0.120)	0.266(0.120)	0.233(0.116)
$\beta_{1,7}$	−0.023(0.128)	−0.098(0.122)	−0.020(0.126)	−0.076(0.117)
$\beta_{1,8}$	0.146(0.130)	0.087(0.125)	0.147(0.127)	0.102(0.121)
$\beta_{2,1}$	−0.356(0.154)	−0.360(0.153)	−0.371(0.153)	−0.351(0.150)
$\beta_{2,2}$	−0.580(0.167)	−0.592(0.163)	−0.559(0.167)	−0.551(0.160)
$\beta_{2,3}$	−0.656(0.179)	−0.683(0.165)	−0.637(0.181)	−0.616(0.162)
$\beta_{2,4}$	−0.716(0.204)	−0.787(0.172)	−0.659(0.204)	−0.703(0.170)
$\beta_{3,1}$	−0.027(0.210)	−0.055(0.203)	0.028(0.210)	−0.054(0.212)
$\beta_{3,2}$	−0.164(0.193)	−0.203(0.202)	−0.191(0.206)	−0.266(0.201)
$\beta_{3,3}$	−0.165(0.126)	−0.168(0.126)	−0.198(0.131)	−0.190(0.131)
$\beta_{3,4}$	0.343(0.128)	0.338(0.130)	0.303(0.131)	0.276(0.131)
$\beta_{3,5}$	−0.121(0.191)	−0.133(0.187)	−0.102(0.201)	−0.112(0.198)
$\beta_{3,6}$	−0.084(0.294)	−0.106(0.291)	−0.258(0.301)	−0.307(0.308)
$\beta_{3,7}$	−0.007(0.157)	−0.035(0.153)	0.068(0.158)	−0.005(0.163)
$\beta_{4,1}$	−0.033(0.099)	−0.043(0.096)	−0.036(0.100)	−0.054(0.100)
$\beta_{4,2}$	0.062(0.177)	0.057(0.172)	0.058(0.179)	0.048(0.182)
$\beta_{5,1}$	−0.017(0.018)	−0.008(0.018)		
$\beta_{5,2}$	0.134(0.018)	0.134(0.018)		
$\beta_{5,3}$	0.039(0.019)	0.037(0.019)		
$\beta_{5,4}$	−0.090(0.023)	−0.092(0.023)		
$\beta_{5,5}$	0.143(0.025)	0.141(0.024)		
$\beta_{5,6}$	0.056(0.021)	0.053(0.021)		
$\beta_{5,7}$	0.205(0.030)	0.211(0.030)		
$\beta_{6,1}$	0.032(0.015)		0.058(0.014)	
$\beta_{6,2}$	−0.007(0.016)		0.000(0.013)	
$\beta_{6,3}$	0.014(0.013)		0.017(0.013)	
$\beta_{6,4}$	0.045(0.081)		0.166(0.076)	
$\beta_{6,5}$	0.021(0.022)		0.017(0.021)	
$\beta_{7,1}$	0.023(0.021)		0.034(0.020)	
$\beta_{7,2}$	0.007(0.026)		0.000(0.025)	
$\beta_{7,3}$	0.031(0.022)		0.042(0.021)	
$\beta_{7,4}$	−0.013(0.017)		−0.019(0.017)	
$\beta_{7,5}$	0.035(0.041)		0.064(0.040)	
$\beta_{7,6}$	−0.041(0.024)		−0.041(0.023)	
$\beta_{7,7}$	−0.021(0.013)		−0.022(0.012)	
$\beta_{8,1}$	−0.058(0.084)		−0.057(0.082)	
$\beta_{8,2}$	0.019(0.019)		0.008(0.019)	
$\beta_{8,3}$	−0.018(0.021)		−0.024(0.020)	
$\beta_{8,4}$	0.040(0.027)		0.030(0.027)	
$\beta_{8,5}$	0.021(0.022)		0.032(0.022)	
$\beta_{8,6}$	0.013(0.039)		0.006(0.038)	
$\beta_{9,1}$	−0.027(0.030)		−0.037(0.030)	
$\beta_{9,2}$	0.015(0.009)		0.009(0.009)	
$\beta_{10,1}$	−0.001(0.007)		0.001(0.006)	
$\beta_{10,2}$	0.002(0.008)		0.002(0.008)	
$\mu_{\alpha }$	−0.137(0.582)	0.421(0.181)	−0.758(0.562)	0.712(0.176)
$\sigma_{\alpha }$	0.934(0.086)	0.927(0.086)	1.065(0.094)	1.099(0.096)
WAIC	3964.87	4083.68	5460.17	5604.75
(**b**)				
β	**Model C1**	**Model C2**	**Model C3**	**Model C4**
$\beta_{1,1}$	−0.260(0.119)	−0.257(0.119)	−0.299(0.116)	−0.290(0.117)
$\beta_{1,2}$	−0.339(0.120)	−0.351(0.118)	−0.351(0.117)	−0.356(0.117)
$\beta_{1,3}$	−0.119(0.123)	−0.203(0.121)	−0.247(0.117)	−0.250(0.119)
$\beta_{1,4}$	−0.365(0.127)	−0.373(0.119)	−0.395(0.123)	−0.391(0.117)
$\beta_{1,5}$	−0.628(0.128)	−0.631(0.122)	−0.596(0.124)	−0.588(0.118)
$\beta_{1,6}$	−0.592(0.131)	−0.586(0.125)	0.528(0.126)	−0.510(0.121)
$\beta_{1,7}$	−0.624(0.135)	−0.639(0.127)	−0.623(0.132)	−0.624(0.124)
$\beta_{1,8}$	−0.575(0.136)	−0.571(0.130)	0.542(0.133)	−0.532(0.126)
$\beta_{2,1}$	0.031(0.158)	0.081(0.156)	0.082(0.156)	0.151(0.152)
$\beta_{2,2}$	−0.232(0.173)	−0.209(0.165)	−0.148(0.172)	−0.091(0.163)
$\beta_{2,3}$	−0.465(0.192)	−0.488(0.171)	−0.284(0.193)	−0.255(0.169)
$\beta_{2,4}$	−0.528(0.217)	−0.579(0.183)	−0.263(0.218)	−0.276(0.180)
$\beta_{3,1}$	−0.219(0.239)	−0.252(0.230)	−0.140(0.236)	−0.200(0.241)
$\beta_{3,2}$	0.172(0.215)	0.117(0.208)	0.009(0.219)	−0.093(0.217)
$\beta_{3,3}$	−0.172(0.137)	−0.167(0.137)	−0.155(0.140)	−0.152(0.139)
$\beta_{3,4}$	−0.179(0.129)	−0.185(0.130)	−0.162(0.133)	−0.175(0.134)
$\beta_{3,5}$	0.064(0.213)	0.060(0.216)	0.026(0.218)	0.018(0.219)
$\beta_{3,6}$	−0.230(0.276)	−0.293(0.274)	−0.183(0.278)	−0.237(0.277)
$\beta_{3,7}$	0.197(0.176)	0.151(0.172)	0.220(0.178)	0.155(0.177)
$\beta_{4,1}$	−0.181(0.106)	−0.185(0.105)	−0.201(0.108)	−0.205(0.108)
$\beta_{4,2}$	0.058(0.170)	0.055(0.173)	0.032(0.179)	0.036(0.173)
$\beta_{5,1}$	−0.057(0.019)	−0.051(0.019)		
$\beta_{5,2}$	0.083(0.017)	0.084(0.017)		
$\beta_{5,3}$	−0.009(0.020)	−0.001(0.019)		
$\beta_{5,4}$	−0.109(0.024)	−0.112(0.024)		
$\beta_{5,5}$	0.126(0.026)	0.119(0.025)		
$\beta_{5,6}$	−0.016(0.022)	−0.017(0.022)		
$\beta_{5,7}$	0.317(0.031)	0.318(0.031)		
$\beta_{6,1}$	0.011(0.015)		0.024(0.015)	
$\beta_{6,2}$	0.008(0.018)		0.012(0.017)	
$\beta_{6,3}$	0.029(0.014)		0.024(0.014)	
$\beta_{6,4}$	0.023(0.079)		0.091(0.078)	
$\beta_{6,5}$	0.028(0.022)		0.035(0.022)	
$\beta_{7,1}$	0.010(0.020)		0.010(0.020)	
$\beta_{7,2}$	−0.027(0.027)		−0.013(0.026)	
$\beta_{7,3}$	0.008(0.022)		0.024(0.021)	
$\beta_{7,4}$	0.018(0.018)		0.031(0.018)	
$\beta_{7,5}$	0.024(0.044)		0.021(0.043)	
$\beta_{7,6}$	0.009(0.025)		0.003(0.025)	
$\beta_{7,7}$	−0.010(0.014)		−0.019(0.013)	
$\beta_{8,1}$	0.162(0.085)		0.127(0.084)	
$\beta_{8,2}$	0.016(0.020)		0.012(0.020)	
$\beta_{8,3}$	0.011(0.021)		0.009(0.021)	
$\beta_{8,4}$	−0.045(0.029)		−0.030(0.029)	
$\beta_{8,5}$	0.030(0.023)		0.040(0.023)	
$\beta_{8,6}$	0.050(0.043)		0.059(0.042)	
$\beta_{9,1}$	0.006(0.032)		−0.006(0.032)	
$\beta_{9,2}$	0.024(0.009)		0.021(0.009)	
$\beta_{10,1}$	−0.011(0.007)		−0.009(0.007)	
$\beta_{10,2}$	−0.008(0.008)		−0.011(0.008)	
$\mu_{\alpha }$	−0.519(0.587)	0.766(0.186)	−0.842(0.573)	0.869(0.184)
$\sigma_{\alpha }$	0.963(0.091)	0.973(0.093)	1.060(0.095)	1.076(0.097)
WAIC	3917.99	4044.79	4884.49	5024.25
(**c**)				
β	**Model D1**	**Model D2**	**Model D3**	**Model D4**
$\beta_{1,1}$	0.162(0.237)	0.136(0.231)	0.088(0.229)	0.074(0.225)
$\beta_{1,2}$	−0.093(0.238)	−0.101(0.232)	−0.109(0.231)	−0.125(0.227)
$\beta_{1,3}$	−0.284(0.242)	−0.343(0.239)	−0.325(0.233)	−0.376(0.227)
$\beta_{1,4}$	−0.183(0.254)	−0.184(0.236)	−0.173(0.244)	−0.206(0.226)
$\beta_{1,5}$	−0.630(0.264)	−0.619(0.246)	0.644(0.253)	−0.679(0.238)
$\beta_{1,6}$	−0.481(0.267)	−0.481(0.248)	−0.472(0.254)	−0.507(0.239)
$\beta_{1,7}$	0.033(0.271)	0.006(0.250)	0.018(0.263)	−0.069(0.242)
$\beta_{1,8}$	−0.134(0.276)	−0.151(0.259)	−0.245(0.267)	−0.320(0.249)
$\beta_{2,1}$	−0.074(0.244)	−0.030(0.235)	−0.049(0.240)	−0.037(0.230)
$\beta_{2,2}$	0.130(0.272)	0.104(0.263)	0.184(0.269)	0.118(0.256)
$\beta_{2,3}$	0.346(0.306)	0.224(0.281)	0.361(0.304)	0.133(0.272)
$\beta_{2,4}$	0.293(0.371)	0.135(0.317)	0.557(0.371)	0.260(0.305)
$\beta_{3,1}$	0.199(0.514)	0.162(0.496)	0.122(0.515)	0.051(0.498)
$\beta_{3,2}$	0.682(0.391)	0.634(0.376)	0.540(0.392)	0.414(0.386)
$\beta_{3,3}$	0.392(0.310)	0.336(0.302)	0.225(0.323)	0.126(0.304)
$\beta_{3,4}$	0.775(0.277)	0.71(0.278)	0.758(0.288)	0.647(0.275)
$\beta_{3,5}$	0.501(0.385)	0.500(0.374)	0.576(0.382)	0.589(0.370)
$\beta_{3,6}$	0.646(0.674)	0.657(0.652)	0.163(0.654)	0.121(0.642)
$\beta_{3,7}$	−0.066(0.384)	−0.071(0.379)	0.219(0.403)	0.064(0.395)
$\beta_{4,1}$	0.002(0.220)	0.021(0.212)	−0.038(0.223)	0.020(0.217)
$\beta_{4,2}$	−0.726(0.418)	−0.724(0.396)	−0.861(0.417)	−0.727(0.398)
$\beta_{5,1}$	−0.054(0.038)	−0.050(0.037)		
$\beta_{5,2}$	0.156(0.037)	0.149(0.035)		
$\beta_{5,3}$	0.018(0.037)	0.016(0.036)		
$\beta_{5,4}$	−0.024(0.040)	−0.028(0.038)		
$\beta_{5,5}$	0.052(0.044)	0.058(0.042)		
$\beta_{5,6}$	0.053(0.042)	0.058(0.042)		
$\beta_{5,7}$	0.238(0.060)	0.029(0.059)		
$\beta_{6,1}$	0.043(0.029)		0.065(0.030)	
$\beta_{6,2}$	0.001(0.037)		−0.003(0.037)	
$\beta_{6,3}$	0.034(0.032)		0.042(0.031)	
$\beta_{6,4}$	−0.040(0.086)		0.027(0.083)	
$\beta_{6,5}$	0.091(0.047)		0.103(0.045)	
$\beta_{7,1}$	−0.008(0.043)		0.038(0.041)	
$\beta_{7,2}$	−0.027(0.061)		−0.012(0.059)	
$\beta_{7,3}$	−0.081(0.039)		−0.062(0.038)	
$\beta_{7,7}$	0.036(0.030)		0.039(0.029)	
$\beta_{8,1}$	−0.197(0.126)		−0.180(0.122)	
$\beta_{8,2}$	0.017(0.044)		0.006(0.043)	
$\beta_{8,3}$	0.031(0.048)		0.025(0.047)	
$\beta_{8,4}$	−0.069(0.060)		−0.040(0.057)	
$\beta_{8,5}$	0.087(0.049)		0.108(0.047)	
$\beta_{8,6}$	−0.042(0.076)		−0.009(0.073)	
$\beta_{9,1}$	−0.072(0.079)		−0.076(0.078)	
$\beta_{9,2}$	−0.001(0.022)		0.006(0.021)	
$\beta_{10,1}$	0.012(0.013)		0.010(0.012)	
$\beta_{10,2}$	0.003(0.016)		0.008(0.015)	
$\mu_{\alpha }$	0.148(0.805)	−0.627(0.303)	−0.719(0.773)	−0.346(0.293)
$\sigma_{\alpha }$	1.252(0.227)	1.241(0.217)	1.380(0.240)	1.356(0.232)
WAIC	607.020	700.744	973.582	1077.95
(**d**)				
β	**Model E1**	**Model E2**	**Model E3**	**Model E4**
$\beta_{1,1}$	−0.142(0.199)	−0.130(0.199)	−0.174(0.194)	−0.157(0.191)
$\beta_{1,2}$	−0.098(0.198)	−0.097(0.195)	−0.115(0.196)	−0.108(0.191)
$\beta_{1,3}$	−0.355(0.198)	−0.344(0.197)	−0.349(0.192)	−0.336(0.190)
$\beta_{1,4}$	−0.428(0.217)	−0.349(0.197)	−0.434(0.211)	−0.351(0.189)
$\beta_{1,5}$	−0.334(0.221)	−0.252(0.204)	−0.276(0.215)	−0.188(0.195)
$\beta_{1,6}$	−0.276(0.222)	−0.212(0.205)	−0.211(0.217)	−0.141(0.198)
$\beta_{1,7}$	−0.425(0.232)	−0.374(0.211)	−0.392(0.222)	−0.329(0.202)
$\beta_{1,8}$	−0.743(0.236)	−0.659(0.215)	−0.700(0.229)	−0.615(0.209)
$\beta_{2,1}$	−0.035(0.261)	−0.014(0.256)	−0.142(0.258)	−0.108(0.255)
$\beta_{2,2}$	−0.088(0.285)	−0.067(0.278)	−0.201(0.285)	−0.154(0.279)
$\beta_{2,3}$	−0.335(0.300)	−0.424(0.275)	−0.221(0.303)	−0.286(0.279)
$\beta_{2,4}$	−0.08(0.311)	−0.219(0.262)	0.117(0.312)	−0.006(0.265)
$\beta_{3,1}$	−0.074(0.456)	−0.103(0.453)	0.181(0.469)	0.108(0.462)
$\beta_{3,2}$	−0.069(0.344)	−0.014(0.345)	−0.209(0.350)	−0.225(0.332)
$\beta_{3,3}$	−0.072(0.260)	0.043(0.256)	−0.086(0.269)	0.004(0.261)
$\beta_{3,4}$	−0.222(0.244)	−0.123(0.241)	−0.249(0.248)	−0.167(0.243)
$\beta_{3,5}$	−0.193(0.346)	−0.166(0.341)	−0.335(0.345)	−0.321(0.340)
$\beta_{3,6}$	−0.011(0.432)	0.046(0.426)	−0.231(0.429)	−0.226(0.438)
$\beta_{3,7}$	0.001(0.268)	0.011(0.262)	0.128(0.275)	0.111(0.273)
$\beta_{4,1}$	0.140(0.189)	0.125(0.184)	0.162(0.190)	0.152(0.184)
$\beta_{4,2}$	0.282(0.318)	0.329(0.306)	0.166(0.315)	0.229(0.309)
$\beta_{5,1}$	0.026(0.030)	0.016(0.029)		
$\beta_{5,2}$	0.116(0.028)	0.113(0.027)		
$\beta_{5,3}$	−0.011(0.031)	−0.008(0.03)		
$\beta_{5,4}$	−0.064(0.041)	−0.067(0.041)		
$\beta_{5,5}$	0.113(0.043)	0.118(0.042)		
$\beta_{5,6}$	0.051(0.036)	0.043(0.036)		
$\beta_{5,7}$	0.149(0.046)	0.152(0.046)		
$\beta_{6,1}$	−0.022(0.024)		−0.008(0.024)	
$\beta_{6,2}$	0.076(0.034)		0.077(0.034)	
$\beta_{6,3}$	0.091(0.031)		0.087(0.031)	
$\beta_{6,4}$	−0.069(0.091)		−0.032(0.089)	
$\beta_{6,5}$	0.003(0.039)		0.002(0.037)	
$\beta_{7,1}$	−0.029(0.033)		−0.011(0.032)	
$\beta_{7,2}$	0.002(0.05)		0.029(0.049)	
$\beta_{7,3}$	−0.046(0.036)		−0.036(0.036)	
$\beta_{7,7}$	−0.006(0.024)		−0.005(0.024)	
$\beta_{8,1}$	0.070(0.115)		0.045(0.113)	
$\beta_{8,2}$	0.011(0.037)		−0.003(0.037)	
$\beta_{8,3}$	0.006(0.041)		−0.008(0.040)	
$\beta_{8,4}$	0.005(0.048)		0.000(0.047)	
$\beta_{8,5}$	−0.015(0.043)		−0.009(0.043)	
$\beta_{8,6}$	0.099(0.065)		0.077(0.064)	
$\beta_{9,1}$	0.070(0.065)		0.050(0.063)	
$\beta_{9,2}$	0.018(0.016)		0.016(0.016)	
$\beta_{10,1}$	−0.006(0.01)		−0.009(0.010)	
$\beta_{10,2}$	−0.018(0.013)		−0.021(0.013)	
$\mu_{\alpha }$	0.236(0.756)	0.237(0.298)	0.249(0.742)	0.337(0.299)
$\sigma_{\alpha }$	1.166(0.182)	1.188(0.178)	1.260(0.183)	1.263(0.182)
WAIC	1346.96	1440.13	1712.62	1804.41

## Data Availability

Restrictions apply to the availability of these data. Data were obtained from Keio University and are available https://www.pdrc.keio.ac.jp/en/ (accessed on 14 January 2025) with the permission of Keio University.

## Abbreviations

The following abbreviations are used in this manuscript:

MCMC	Markov chain Monte Carlo
ASIS	ancillarity–sufficiency interweaving strategy
WAIC	widely applicable information criterion

## Appendix A

### Appendix A.1. Full Conditional of **α**

Applying Bayes’ theorem and the square completion formula, the full conditional of α is derived from Equations (8) and (25) as follows:

(A1) $$\begin{array}{cc} p(\alpha |\theta_{-\alpha },D)\; & \propto p(y|X,\omega ,\alpha ,\beta )p(\alpha )\\ \; & \propto \exp\left[-\frac{1}{2}\alpha^{\prime }V\alpha +{\left(K-Z\beta \right)}^{\prime }\alpha -\frac{1}{2\sigma_{\alpha }^{2}}{\left(\alpha -\mu_{\alpha }\iota \right)}^{\prime }\left(\alpha -\mu_{\alpha }\iota \right)\right]\\ \; & \propto \exp\left[-\frac{1}{2}\alpha^{\prime }\left(V+\sigma_{\alpha }^{-2}I\right)\alpha \right.+{\left(K-Z\beta +\mu_{\alpha }\sigma_{\alpha }^{-2}\iota \right)}^{\prime }\alpha ]. \end{array}$$

This is mathematically equivalent to Equation (26).

### Appendix A.2. Full Conditional of **β**

Applying Bayes’ theorem and the square completion formula, the full conditional of β is derived from Equations (8) and (25) as follows:

(A2) $$\begin{array}{cc} p(\beta |\theta_{-\beta },D)\; & \propto p(y|X,\omega ,\alpha ,\beta )p(\beta )\\ \; & \propto \exp\left[-\frac{1}{2}\beta^{\prime }X^{\prime }\Omega X\beta +{\left(\bar{x}-Z^{\prime }\alpha \right)}^{\prime }\beta -\frac{1}{2}{\left(\beta -\mu_{\beta }\right)}^{\prime }A_{\beta }\left(\beta -\mu_{\beta }\right)\right]\\ \; & \propto \exp\left[-\frac{1}{2}\beta^{\prime }\left(X^{\prime }\Omega X+A_{\beta }\right)\beta \right.+{\left(\bar{x}-Z^{\prime }\alpha +A_{\beta }\mu_{\beta }\right)}^{\prime }\beta ]. \end{array}$$

This is mathematically equivalent to Equation (27).

### Appendix A.3. Full Conditional of μ_α_

Applying Bayes’ theorem and the square completion formula, the full conditional of $\mu_{\alpha }$ is derived from Equations (8) and (9) as follows:

(A3) $$\begin{array}{cc} p(\mu_{\alpha }|\theta_{-\mu_{\alpha }},D)\; & \propto p\left(\alpha |\theta_{-\alpha }\right)p\left(\mu_{\alpha }\right)\\ \; & \propto \exp\left[-\frac{\sum_{r=1}^{R}{\left(\alpha_{r}-\mu_{\alpha }\right)}^{2}}{2\sigma_{\alpha }^{2}}-\frac{{\left(\mu_{\alpha }-\mu_{0}\right)}^{2}}{2\sigma_{0}^{2}}\right]\\ \; & \propto \exp\left[-\frac{1}{2}\{\left(\sigma_{\alpha }^{-2}R+\sigma_{0}^{-2}\right)\mu_{\alpha }^{2}-2\left(\sigma_{\alpha }^{-2}\sum_{r=1}^{R}\alpha_{r}+\sigma_{0}^{-2}\mu_{0}\right)\mu_{\alpha }\right\}] \end{array}$$

(A4) $$\begin{array}{cc} \; & \propto \exp\left[-\frac{1}{2}\left(\sigma_{\alpha }^{-2}R+\sigma_{0}^{-2}\right){\left(\mu_{\alpha }-\frac{\sigma_{\alpha }^{-2}\sum_{r=1}^{R}\alpha_{r}+\sigma_{0}^{-2}\mu_{0}}{\sigma_{\alpha }^{-2}R+\sigma_{0}^{-2}}\right)}^{2}\right]. \end{array}$$

This is mathematically equivalent to Equation (28).

### Appendix A.4. Full Conditional of $\sigma_{\alpha }^{2}$

Applying Bayes’ theorem and the square completion formula, the full conditional of $\sigma_{\alpha }^{2}$ is derived from Equations (8) and (9) as follows:

(A5) $$\begin{array}{cc} p(\sigma_{\alpha }^{2}|\theta_{-\sigma_{\alpha }^{2}},D)\; & \propto p\left(\alpha |\theta_{-\alpha }\right)p\left(\sigma_{\alpha }^{2}\right)\\ \; & \propto {\left(\sigma_{\alpha }^{2}\right)}^{-\frac{R}{2}}\exp\left[-\frac{\sum_{r=1}^{R}{\left(\alpha_{r}-\mu_{\alpha }\right)}^{2}}{2\sigma_{\alpha }^{2}}\right]{\left(\sigma_{\alpha }^{2}\right)}^{-\frac{\nu_{0}}{2}+1}\exp\left[-\frac{\lambda_{0}}{2\sigma_{\alpha }^{2}}\right]\\ \; & \propto {\left(\sigma_{\alpha }^{2}\right)}^{-\frac{R+\nu_{0}}{2}+1}\exp\left[-\frac{\sum_{r=1}^{R}{\left(\alpha_{r}-\mu_{\alpha }\right)}^{2}+\lambda_{0}}{2\sigma_{\alpha }^{2}}\right]. \end{array}$$

This is mathematically equivalent to Equation (29).

### Appendix A.5. Gibbs Sampling and Efficiency Improvement Using ASIS

The Gibbs sampling procedure employed for the panel data logit model is as follows:

This study involved estimating a large number of parameters, and the sampled random number sequence exhibited high autocorrelation even with a long lag. To improve the efficiency of Gibbs sampling, we implemented the ASIS algorithm (Yu & Meng, 2011). ASIS alternates between sampling from models with and without centralization of latent variables. Despite its simplicity, it is a highly efficient algorithm. Although ASIS is most commonly used for estimating state-space models, it can also be applied to panel data models (e.g., Nakakita & Nakatsuma, 2023; Saito et al., 2024).

Next, we applied noncentered parameterization:

(A6) $$\begin{array}{c} \psi_{rt}=\alpha_{r}+x_{rt}^{\prime }\beta ,\;\alpha_{r}∼N\left(\mu_{\alpha },\sigma_{\alpha }^{2}\right). \end{array}$$

Additionally, we considered centered parameterization:

(A7) $$\begin{array}{cc} \tilde{\alpha_{r}} & =\alpha_{r}-\mu_{\alpha }, \end{array}$$

(A8) $$\begin{array}{cc} \psi_{rt} & =\tilde{\alpha_{r}}+\mu_{\alpha }+x_{rt}^{\prime }\beta ,\;\tilde{\alpha_{r}}∼N\left(0,\sigma_{\alpha }^{2}\right). \end{array}$$

To modify the full conditionals into centered parameterization, we replaced ($x_{rt},\alpha ,\beta )$ with

(A9) $$\begin{array}{c} {\tilde{x}}_{rt}=\left[\begin{array}{c} 1\\ x_{rt} \end{array}\right],\;\tilde{\alpha }=\left[\begin{array}{c} {\tilde{\alpha }}_{1}\\ \vdots \\ {\tilde{\alpha }}_{R} \end{array}\right],\;\tilde{\beta }=\left[\begin{array}{c} \mu_{\alpha }\\ \beta \end{array}\right], \end{array}$$

and ($X,\bar{x},Z$) with

(A10) $$\begin{array}{c} \tilde{X}={\left[\begin{array}{c} {\tilde{x}}_{11}^{\prime }\ldots {\tilde{x}}_{1T_{1}}^{\prime }\ldots {\tilde{x}}_{R1}^{\prime }\ldots {\tilde{x}}_{RT_{R}}^{\prime } \end{array}\right]}^{\prime },\;\tilde{\bar{x}}=\sum_{r=1}^{R}\sum_{t=1}^{T_{r}}\kappa_{rt}{\tilde{x}}_{rt},\;\tilde{Z}=\left[\begin{array}{c} \sum_{t=1}^{T_{1}}\omega_{1t}{\tilde{x}}_{1t}^{\prime }\\ \vdots \\ \sum_{t=1}^{T_{R}}\omega_{Rt}{\tilde{x}}_{Rt}^{\prime } \end{array}\right], \end{array}$$

setting the prior distribution of $\tilde{\beta }$ as

(A11) $$\begin{array}{c} \;\tilde{\beta }∼N\left({\tilde{\mu }}_{\beta },{\tilde{A}}_{\beta }^{-1}\right),\;{\tilde{\mu }}_{\beta }=\left[\begin{array}{c} \mu_{0}\\ \mu_{\beta } \end{array}\right],\;{\tilde{A}}_{\beta }=\left[\begin{array}{cc} \sigma_{0}^{-2} & \\ \; & A_{\beta } \end{array}\right], \end{array}$$

with $\mu_{\alpha }=0$ in the full conditional of α and $\sigma_{\alpha }^{2}$. Notably, β and $\mu_{\alpha }$ were generated together from the full conditional of $\tilde{\beta }$ in centered parameterization.

Applying the same replacements in Equations (16) and (27)–(29), the full conditionals of parameters $(\tilde{\beta }=\left[\mu_{\alpha };\beta \right],\omega_{rt},$$\sigma_{\alpha }^{2})$ were derived as follows:

(A12) $$\begin{array}{cc} \tilde{\beta }|\theta_{-\tilde{\beta }},D & ∼N\left({\left({\tilde{X}}^{\prime }\Omega \tilde{X}+{\tilde{A}}_{\beta }\right)}^{-1}\left(\tilde{\bar{x}}-{\tilde{Z}}^{\prime }\tilde{\alpha }+{\tilde{A}}_{\beta }{\tilde{\mu }}_{\beta }\right),\right.{\left({\tilde{X}}^{\prime }\Omega \tilde{X}+{\tilde{A}}_{\beta }\right)}^{-1}), \end{array}$$

(A13) $$\begin{array}{cc} \;\omega_{rt}|\theta_{-\omega },D & ∼\mathrm{PG}(1,\tilde{\alpha_{r}}+{\tilde{x}}_{rt}^{\prime }\tilde{\beta }), \end{array}$$

(A14) $$\begin{array}{cc} \;\sigma_{\alpha }^{2}|\theta_{-\sigma_{\alpha }^{2}},D & ∼\mathrm{IG}\left(\frac{R+\nu_{0}}{2},\frac{\sum_{r=1}^{R}{\tilde{\alpha_{r}}}^{2}+\lambda_{0}}{2}\right). \end{array}$$

Therefore, the Gibbs sampling procedure with ASIS may be summarized as follows:
